# Individual vocal identity is enhanced by the enlarged external nose in male proboscis monkeys (*Nasalis larvatus*)

**DOI:** 10.1098/rsif.2025.0098

**Published:** 2025-08-13

**Authors:** Tomoki Yoshitani, Rintaro Miyazaki, Satoru Seino, Kazuya Edamura, Koichi Murata, Ikki Matsuda, Takeshi Nishimura, Isao T. Tokuda

**Affiliations:** ^1^ Graduate School of Science and Engineering, Ritsumeikan University, Kusatsu, Shiga, Japan; ^2^ Preservation and Research Center, City of Yokohama, Yokohama, Kanagawa, Japan; ^3^ Graduate School of Bioresource Sciences, Nihon University, Fujisawa, Kanagawa, Japan; ^4^ Yokohama Zoological Gardens ‘ZOORASIA’, Yokohama, Kanagawa, Japan; ^5^ Wildlife Research Center, Kyoto University, Sakyo, Kyoto, Japan; ^6^ Chubu University, Kasugai, Aichi, Japan; ^7^ Institute for Tropical Biology and Conservation, Universiti Malaysia Sabah, Kota Kinabalu, Sabah, Malaysia; ^8^ Graduate School of Human Sciences, The University of Osaka, Suita, Osaka, Japan

**Keywords:** bioacoustics, formant, multilevel sociality, patrilineal community, nasal passage

## Abstract

Adult male proboscis monkeys, *Nasalis larvatus*, develop an enlarged external nose. Males often produce loud, long-distance calls filtered through the nasal passage. The enlarged nose probably functions as a visual badge of social status and a visual key representing the owner’s physical and sexual quality, and thus is useful for females in selecting mates. In addition to such visual signalling, a larger external nose enhances the lower frequencies in calls, possibly exaggerating acoustic signals related to body size. Here, we used computational simulations with three-dimensional models of the nasal passage to show how the external nose modifies the acoustic property, indicating that the external nose develops to enhance lower frequencies in adults but varies in a specific formant position among adult males. This finding suggests that the external nose generates acoustic signals about physical–sexual maturity in adult males and individual identity among them. The unusual features of the social organization in this species, a patrilineality of a multilevel community consisting of one-male–multi-female units, may reinforce the functional importance of individual male recognition for males and females to monitor the location of both their own units and those of other males.

## 1. Introduction

Vocal acoustics transmit biological signals of the vocalizer: body size, sex, developmental stage, including reproductive status, and individual identity. Human voices are generated in a two-step process: a vocal source is produced in the glottis and a spectral structure is added to the source by filtering through the vocal tract and nasal passage [1,2]. The bands of frequencies amplified by filtering are usually expressed in ascending order, such as the first and second formants (abbreviated as F1 and F2). In humans, while the positions of lower formants like F1 and F2 are critical for vowel identification in speech [1,2], higher formants like F3 and F4 often play a role in signalling an individual’s identity [3,4]. The laryngeal cavity contributes, in part, to individual variation in the F3 position [5]. The cavity is located within the larynx framed by the laryngeal cartilages. While the cavity shape varies between individuals, its topology does not change much during vocalization [5,6]. This anatomical property adds a constant signal of individuality to a voice.

Adult male proboscis monkeys, *Nasalis larvatus*, are known for their enlarged external noses. The enlarged nose probably functions as a visual badge of social status, reducing the need for physical confrontation between males [7,8], and as a visual signal for females by representing the physical and sexual quality of a male [9]. This species is characterized by a core reproductive unit of one-male–multi-female units (OMUs) and a higher-level community—so-called multilevel socialites (MLS)—where an OMU regularly aggregates with other OMUs and bachelor groups [10,11]. Such a social organization has potentially high male-to-male competition to drive the evolution of such a distinctive precopulatory visual display for female choice in mate selection [12]. One of the most compelling parallels can be found in the African mandrills, which form OMU despite being a massive single-layered group, and sport the most colourful males among primates [13]. In addition, the large nose in adult male proboscis monkeys may exaggerate an acoustic signal of body size, thus serving the dual purpose of deterring potential rivals and attracting mates [7]. Males often produce a loud, long-distance call, termed a ‘bray’, which is filtered through the nasal passage and emitted while the mouth is closed [14]. In brays from a male with a larger external nose, the F3 position shifts downward relative to the lower formants [7]. Such acoustic modifications could theoretically have the effect of exaggerating signals about the vocalizer’s body size, although the effect is yet to be experimentally examined [7,15,16]. The internal tract of the nasal passage lacks muscular apparatus that actively drives their topological modifications and does not change as much as the vocal tract that is modified by tongue and the other skeletal and muscular organs, during vocalization, so individual-specific variation in a specific formant may also serve as a constant signal of individual identity.

Here, we used computational simulations with three-dimensional models of the nasal passage to investigate how the external nose modifies the positions of formants in male proboscis monkeys. We show that the external nose develops to emphasize low frequencies in adult males compared with juvenile ones and that the size of the external nose alters specifically the F3 position among adult males, suggesting that this distinctive feature generates signals of physical maturity and individual identity in adults. Finally, we discuss how the specific social organization of this species reinforces the functional importance of individual male recognition for social communication.

## 2. Results

**Figure 1.**
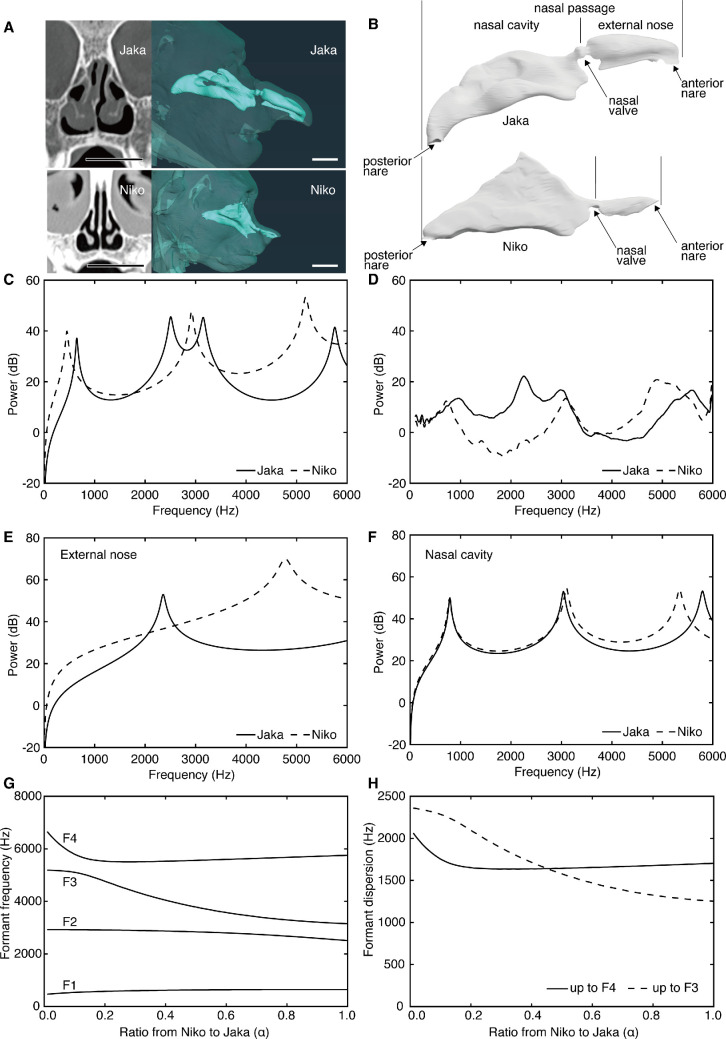
Three-dimensional models, area functions and calculated transfer functions of the nasal passage. (A) Coronal CT scans (the left side of the nasal passage, the right side on the screen, is used here) and the nasal passage within the head and external nose (upper, adult Jaka; lower, juvenile Niko), scale 2 cm; (B) three-dimensional models reconstructed from CT scans; (C) transfer functions calculated from the area functions in (B); (D) transfer functions measured experimentally from the replica model; (E, F) transfer functions of the nasal cavity (E) and external nose (F) extracted from the area functions in (B); (G, H) formants (G) and formant dispersion parameters (Dfs) (H) estimated by morphing the area function in (B) from juvenile Niko to adult Jaka.

We used computational models to show that F3 is shifted downward by the development of a large external nose. Computed tomographic (CT) scans were taken post-mortem from two male individuals, the adult Jaka (13 years old, 17 kg: maximum weight when healthy before death) and the juvenile Niko (3 years old, 4 kg), which were housed at the Yokohana Zoological Gardens ‘ZOORASIA’ (figure 1A) [17]. The scans were used to generate three-dimensional surface data of the nasal passage from the posterior to the anterior nares (figure 1B). The cross-sectional area function was drawn along the centre line of the nasal passage following methods outlined in [6] (electronic supplementary material, figure S1A,B). The area drops markedly at the nasal valve (figure 1B and electronic supplementary material, figure S1C). Hereafter, the regions posterior and anterior to the nasal valve are referred to as the nasal cavity and the external nose, respectively (figure 1B). This means that the two regions can function acoustically as independent filters [1,2]. To examine the acoustic effects of the external nose, the scale of the nasal passage for juvenile Niko was enlarged to make the length of the nasal cavity the same as that of the adult Jaka. The external nose was proportionally larger and longer in the adult than in the juvenile specimen (figure 1B and electronic supplementary material, figure S1C). The first four formants were computationally calculated from the cross-sectional area functions: the positions of F1 and F2 were almost the same between the adult and juvenile specimens (table 1 and figure 1C); F3 was close to F2 in the adult; F4 in the adult almost corresponded to F3 in the juvenile; F4 was positioned slightly higher than F3 in the juvenile.

**Table 1.** Formant frequencies (Hz) estimated from the numerical method and the replica experiments.

method	individual	F1	F2	F3	F4
numerical	Jaka	645	2505	3150	5755
	Niko	450	2920	5180	6655
replica	Jaka	961	2253	2979	5575
	Niko	714	3081	4902	—

We used physical models generated with the geometric data used for the computational calculations to experimentally confirm the differences in formant distribution between the adult and juvenile specimens. The acoustic property of the nasal passage was measured following methods outlined in [18]. The frequency of the sweep tone sound inputted into the nasal passage was slowly increased from 1 to 6000 Hz, and the transfer functions were measured. Although slight differences were observed in the peak positions that corresponded to the formant positions, the experimental results were in good agreement with the estimates from the computational models (figure 1D; average absolute differences: 230 Hz for adult Jaka, and 234 Hz for juvenile Niko). Thus, the computational models used here estimate the filtering property of the nasal passage well.

To investigate how the external nose size influences the filtering property of the nasal passage, the transfer function was computationally calculated separately for the regions of the nasal cavity and external nose for the two specimens following methods outlined in [18]. A large difference was found for the external nose (figure 1E), while the formant positions were similar for the nasal cavity (figure 1F). For the external nose, the position of F1 was more than 2400 Hz lower in the adult compared with the juvenile (Jaka: 2355 Hz, Niko: 4775 Hz; figure 1E). Next, to see the effect of the external nose size on the acoustic property, we calculated the filtering property of the entire nasal passage by smoothly changing the cross-sectional area function from juvenile Niko to adult Jaka (electronic supplementary material, figure S2). This morphing mimics the developmental change in filtering property from juvenile to adult. Consequently, F3 continued to shift downward to be close to F2 (figure 1G). F4 shifted downward before the 20% point of the change from juvenile Niko to adult Jaka, but then stabilized close to the original position of F3 (figure 1G). Thus, the external nose size is the main determinant of changes in F3 and F4 positions between juveniles and adults and of changes in the F3 position among adults.

To assess the effects of the formant changes on the acoustic signal, formant dispersion parameters (Dfs) were examined using the estimates from the computational models. Df is the average distance between each adjacent pair of formants and is theoretically negatively correlated with body size, which is represented by the length of the filter, namely, the vocal tract [15]. Here, we consider the nasal passage as a filter. Df for F1 up to F3 (DfF3) was 1113 Hz smaller in the adult compared with the juvenile specimens (Jaka: 1253 Hz, Niko: 2365 Hz). This is because F3 was much lower in the adult than in the juvenile (table 1, figure 1C and 1G). However, Df for F1 up to F4 (DfF4) was only 365 Hz smaller (Jaka: 1703 Hz, Niko: 2068 Hz), because F4 was not as different between the two individuals (table 1). In the morphing that mimicked the developmental changes, DfF4 decreased to reach the adult level before the 20% point from juvenile Niko to adult Jaka and did not change thereafter (figure 1H). Thus, the external nose develops to change the acoustic signal about body size between juveniles and adults. Few changes of DfF4 after the 20% point, however, imply that the external nose does not add noticeable variability in the signal about body size among adults in male proboscis monkeys.

## 3. Discussion

Our findings support the view that adult males develop the external nose to exaggerate a signal of body size to female partners and immature males [7]. In the MLS of proboscis monkeys, the dynamics of male–male competition and female choice should potentially be pronounced due to the frequent spatial overlap of multiple OMUs and bachelor groups [19,20]. This close proximity facilitates frequent male interactions, allowing for competitive displays without direct conflict. In contrast, females frequently transfer between OMUs even after reaching adulthood [9], increasing the role of female choice in selecting mates. The enlarged external nose serves as a visual display and simultaneously as a vocal proxy of body size. These are important signals to distinguish immature from mature males, especially for avoiding unexpected encounters with bachelor groups comprising mostly immature males [7]. Thus, in addition to being a visual signal, the current study supported the view that the enlarged nose may produce an acoustic signal of adulthood (body size reflecting physical and sexual maturity).

However, we showed that variation in external nose size makes a limited contribution to modify an acoustic signal of body size as a marker of physical and sexual superiority between adult males. Alternatively, the external nose may produce an acoustic signal of individual identification with individual variation in the F3 position among adult males. Human speech is individualized by a variety of acoustic features, including the position and temporal variation of pitch (Fo), formants, intensity position and temporal pattern of speaking [3,4,21,22]. Humans produce long and sequential voices when speaking. In contrast, most non-human primates, including proboscis monkeys, typically produce a single call, and therefore individual variation in a specific range of formants is one of the candidates for adding individuality to a call. Humans have a long pharyngeal–laryngeal space, where the laryngeal cavity produces individual variability in a specific formant [5,6]. In contrast, the pharyngeal–laryngeal space in non-human primates is limited [23–25], and the cavities in this space have limited filtering capacity. Thus, the enlarged external nose may be alternatively used to exaggerate vocal individual identification for adult male proboscis monkeys within a MLS community.

Small community size of the band allows individuals to use information about individual identity for social communication in non-human primates. Primates with MLS often form larger communities compared with single-layered primate species [26]. African large papionins often form an MLS structure with the largest communities among primates [27]. In geladas (*Theropithecus gelada*), for instance, the number of males can exceed 30 in a band consisting of OMUs, and further 100 at the higher level of a herd [26,28,29]. As such, in a social situation, it is cognitively difficult to track the social information for individual recognition [13,30], as predicted by the social complexity hypothesis [31,32]. In contrast, proboscis monkeys usually form a band of approximately 10 adult males and a higher-level unit of fewer than 40 males, including the bachelor group [10,33]. In such a small community, recognizing each individual male is less costly, supporting the possibility that an additional faculty of individual identification has evolved in this animal, even in the MLS.

Signals of individual identity are probably reinforced by unique features of the MLS in proboscis monkeys. Specifically, proboscis monkey societies are based on a patrilineal structure, where the genetic patrilineal basis is maintained at the community level [10]. This means that male rather lives within the natal community of bands in MLS, but females disperse from the natal band, although both males and females disperse from the natal OMU, a minimum reproductive unit [9,34]. Adult males that are more closely related to each other would face less competition for females than less related males. Less competitive males can easily aggregate and sleep together in a tree, facilitating efficient defence against nocturnal predators [35,36]. Adult males may have advantages in vocally identifying the location of closely related males’ OMUs while foraging and travelling in dense forests with limited visibility. Such vocal identification may also be relevant for females. In their MLS, females have been observed to transfer between OMUs relatively frequently compared with males [9,34]. When transferring between OMUs, females may vocally identify each male in each OMU and monitor the location of their identified and other males’ OMUs. Thus, the unique patrilineal MLS organization of proboscis monkeys appears to have further driven the evolution of this distinctive nasal feature with enhanced individual identity recognition for social communication.

We strongly support the hypothesis of a dual function: visual and acoustic signalling through the enlarged external nose to aid communication in dense forest environments [7,37]. However, crucial aspects remain unsolved: how these traits elicit any behavioural responses. Empirical investigations, e.g. playback experiments and behavioural studies, are expected to reveal the behavioural responses of females and males to specific visual and acoustic signals from the enlarged external nose of adult males. Such combined approaches would elucidate the adaptive significance of this unique morphology in social communication, including male competitive interactions, reproductive success and social cohesion in proboscis monkeys.

## 4. Methods

### 4.1. Computed tomographic scanning and models

Two male proboscis monkeys, Jaka and Niko, died of natural causes at Yokohama Zoological Gardens, ‘ZOORASIA’, on 29 November 2015 and 20 December 2015, respectively. The cadavers were preserved at the zoo following autopsy. With the zoo’s permission (permission no. RKY 192), frozen specimens from two male proboscis monkeys were thawed and scanned with a 320-row area detector CT scanner (Aquilion One^TM^, Canon Medical Systems, Co., Otawara, Japan) at Nihon University, Fujisawa, Japan, in 2016. The soft palate, nasal cavity and external nose were intact, and the topological deformation can be regarded as minimal (figure 1A), while the tongue, larynx and their associated tissues had been removed at autopsy. The CT data were transformed into a three-dimensional surface image in stereolithography format [38]. The left and right nasal cavities of each proboscis were computationally separated, and the side with less water and tissues was used for study after smoothing rough surfaces.

### 4.2. Transfer function

From the cross-sectional area function, the transfer function of the nasal passage was computed based on the transmission line model [38,39]. By denoting the pressure and volume velocity at the input (the posterior nostril) as *P*_in_ and *U*_in_, respectively, and those at the output (the external nostril) as *P*_out_ and *U*_out_, respectively, their relation can be expressed by a chain matrix as


(4.1) $$\begin{array}{c} \left(\begin{array}{c} P_{out}\\ U_{out} \end{array}\right)=\left(\begin{array}{cc} A_{nasal} & B_{nasal}\\ C_{nasal} & D_{nasal} \end{array}\right)\left(\begin{array}{c} P_{in}\\ U_{in} \end{array}\right) \end{array}.$$

In this modelling, the nasal passage is considered a cascade connection of *N* uniform cylinder tubes with a length of Δ*l*. For the *n*th cylinder (*n* = 1, 2, 3, …, *N*), the input–output relation can be described in the same way as equation (4.1), where the chain matrix and its elements are given by


(4.2) $$\begin{array}{c} K_{n}=\left(\begin{array}{cc} A_{n} & B_{n}\\ C_{n} & D_{n} \end{array}\right) \end{array},$$

(4.3) $$\begin{array}{c} \begin{array}{cc} A_{n}=\cosh\left(\frac{\sigma \Delta l}{c}\right), & B_{n}=-\;\;\frac{\rho c}{a_{n}}\gamma \sinh\left(\frac{\sigma \Delta l}{c}\right),\\ C_{n}=\;–\frac{a_{n}}{\rho c}\frac{1}{\gamma }\sinh\left(\frac{\sigma \Delta l}{c}\right), & D_{n}=\cosh\left(\frac{\sigma \Delta l}{c}\right). \end{array} \end{array}$$

Here, *a*_*n*_ represents the *n*th cross-sectional area, *c* is the sound velocity and *ρ* is the air density. *γ* and *σ* are given by


(4.4) $$\begin{array}{c} \gamma =\sqrt{\frac{r\;+\;j\omega }{\beta +\;j\omega }} \end{array},$$

(4.5) σ=γ(β+jω),

where ω is the angular frequency of the sound wave (ω = 2 π*f* and *f* is frequency). Supposing a rigid wall, *r* and *β* can be set to 0; thus, *γ* = 1，*σ* = *j*ω.

The chain matrix (4.1) of the entire nasal cavity can be computed as a product of the cascaded chain matrices as


(4.6) $$\begin{array}{c} K_{nasal}=\;K_{N}K_{N-1}\cdots \;K_{2}K_{1}=\left(\begin{array}{cc} A_{nasal} & B_{nasal}\\ C_{nasal} & D_{nasal} \end{array}\right) \end{array}.$$

The output pressure is written as the output volume velocity through the radiation impedance at the external nose,


(4.7) $$\begin{array}{c} P_{out}=\;Z_{L}U_{out} \end{array},$$

where the radiation impedance is given by


(4.8) $$\begin{array}{c} Z_{L}=\;\frac{j\omega RL}{R+j\omega L}=\;\frac{\omega^{2}RL^{2}+\;j\omega R^{2}L}{R^{2}+\;\omega^{2}L^{2}} \end{array}$$

with $R=\;\;\frac{128Z_{M}}{9\pi^{2}},\;L=\;\;\frac{8bZ_{M}}{3\pi c},\;b=\;\sqrt{\frac{A_{M}}{\pi }}$ and $Z_{M}=\frac{\rho c}{A_{M}}$, where *b*, *A*_M_ and *Z*_M_ represent the equivalent radius, cross-sectional area and acoustic impedance at the external nose, respectively [40]. Finally, the transfer function is calculated as the ratio of the output pressure to the input volume velocity. By inserting equation (4.7) into equation (4.1) and removing *U*_out_, the transfer function is obtained as


(4.9) $$\begin{array}{c} \frac{P_{out}}{U_{in}}=\;\;\frac{A_{nasal}D_{nasal}-\;B_{nasal}C_{nasal}}{A_{nasal}-\;C_{nasal}Z_{L}}Z_{L} \end{array}.$$

All numerical calculations were performed using MATLAB software (R2020a; MathWorks, Natick, USA).

### 4.3. Measuring the transfer functions of the replica models

**Figure 2.**
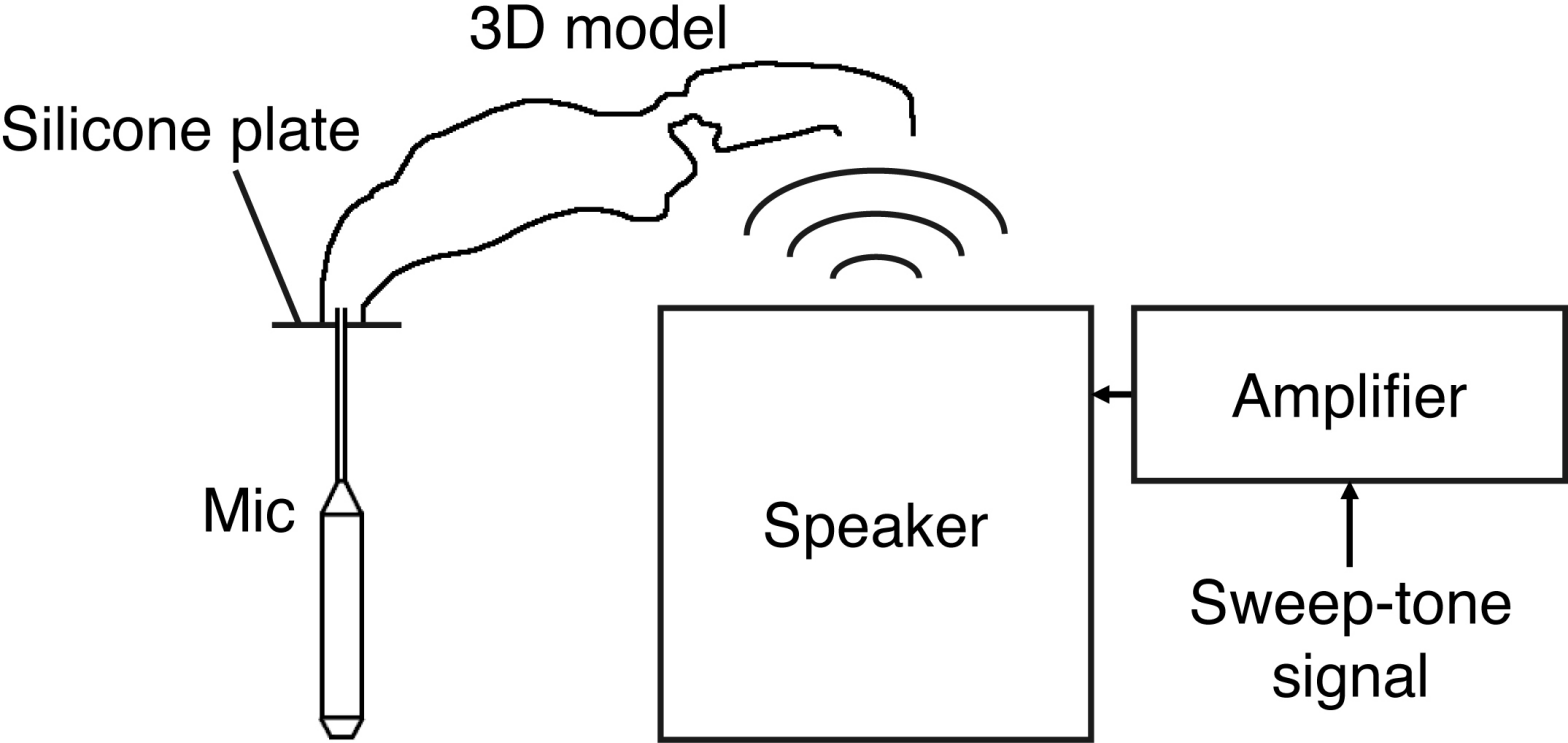
Experimental set-up for measuring the transfer function of the replica model.

The acoustic properties of a replica model of the nasal passage were measured based on the method of [18]. Figure 2 shows the experimental set-up. The models were based on the geometric data used for computational estimation and created at the Center for the Evolutionary Origins of Human Behavior, Kyoto University, by a three-dimensional printer using acrylonitrile–butadiene–styrene resin. A sweep tone sound was generated from a loudspeaker (W3-881SJ; TB Speaker, Taipei, Taiwan) with an amplifier (PC200USB-HR; Fostex, Tokyo) and was inputted from the external nostril side. The distance between the loudspeaker and the external nose was set to 30 mm. The nasopharyngeal side was covered with a silicone plate (Smooth-Sil 940; Smooth-On, Macungie, PA, USA), into which a probe microphone (Type 4182; Brüel & Kjaer, Naerum, Denmark) was inserted through a small hole and linked to a Nexus conditioning amplifier (Brüel & Kjaer). By covering the output, direct injection of the source sound into the microphone was avoided. Acoustic effects below the posterior nostril, such as the ventricle and the vocal tract, were also avoided so that only the acoustic effect of the nasal passage was measured.

Before measurement, the sweep tone sound was recorded in the absence of the nasal passage. To minimize environmental effects (e.g. frequency response and directivity of the microphone, reverberation and room reflection), the input signal was corrected so that the sweep tone sound was generated with equal power at all frequencies.

The corrected signal was applied to the nasal passage, and the output sound was recorded by a digital recorder with a sampling frequency of 44.1 kHz. By applying the Fast Fourier transform to the recorded signal, the transfer function was derived. Because the speaker’s frequency range was between 100 and 20 000 Hz, only the data above 100 Hz were analysed.

### 4.4. Effect of the external nose

To calculate the transfer function of the external nose only, the point of the minimum area was detected, and the area from the minimum point to the external nostril was calculated (electronic supplementary material, figure S1C). The transfer functions were then calculated from the cross-sectional area functions of the external nose and the nasal cavity.

Next, we gradually changed the cross-sectional area function of the whole nasal passage from juvenile Niko to adult Jaka and studied how the transfer function was affected. Because the size of the external nose was significantly different between the two, these morphing analyses can show the effect of the external nose size on the acoustical property. In this morphing, the number of cascaded elements should be equal for the two. Because Niko’s nasal passage was shorter with fewer cross-sections than Jaka’s, spline interpolation was applied so that Niko had the same number of cross-sections as Jaka. Denoting their cross-sectional areas as *A*_JAKA_ and *A*_NIKO_, and positions of the centre line of the cross-section as *d*_JAKA_ and *d*_NIKO_, the area function was calculated for all sections as


(4.10) $$\begin{array}{c} A=\;\alpha A_{JAKA}+\;\left(\alpha -1\right)A_{NIKO} \end{array},$$

(4.11) $$\begin{array}{c} d=\;\alpha d_{JAKA}+\;\left(\alpha -1\right)d_{NIKO} \end{array},$$

where *α* is the weight constant changed from *α* = 0 (Niko) to *α* = 1 (Jaka).

To compute the transfer function, cross-sections must be extracted with equal intervals. Because the cross-sectional intervals were different between the external nose and the nasal cavity, the area function was interpolated again to equalize the intervals.

## Data Availability

All relevant data and resources can be found within the article and its supplementary information. The CT scans, stl data of three-dimensional model and acoustic property calculated at [41]. Supplementary material is available online [42].
